# Diagnostic value of peripheral TiM-3, NT proBNP, and Sestrin2 testing in left-to-right shunt congenital heart disease with heart failure

**DOI:** 10.1186/s12887-022-03823-z

**Published:** 2023-01-04

**Authors:** Qianqian Wang, Guotao Liu, Yan Teng, Xing Feng, Zhiyun Chen, Fen Wang, Yuan Gu, Lishan Jia, Ji Jun Cao, Zhong Xing Lu

**Affiliations:** 1 Department of Neonatology, Taicang Affiliated Hospital of Soochow University (The First People’s Hospital of Taicang), Suzhou City, 215413 Jiangsu Province China; 2 Department of Ultrasonography, Taicang Affiliated Hospital of Soochow University (The First People’s Hospital of Taicang), Suzhou City, 215413 Jiangsu Province China; 3 Department of Pediatrics, Taicang Affiliated Hospital of Soochow University (The First People’s Hospital of Taicang), Suzhou City, 215413 Jiangsu Province China; 4 Department of Pediatrics, Children’s Hospital of Soochow University, Suzhou City, 215002 Jiangsu Province China; 5 Department of Clinical Laboratory, Taicang Affiliated Hospital of Soochow University (The First People’s Hospital of Taicang), Suzhou City, 215413 Jiangsu Province China; 6 Department of Changzhou Maternal and Child Health Care Hospital, Changzhou City, 213003 Jiangsu Province China

**Keywords:** Tim-3, NT-proBNP, Sestrin2, Left-to-right shunt type congenital heart disease, Heart failure, Diagnostic value

## Abstract

**Background:**

Left-to-right shunt congenital heart disease is more likely to induce recurrent respiratory infections in the patients which exacerbate pulmonary hypertension and thereby impairs cardiac function. It is urgent to explore a non-invasive and accurate diagnostic method that can show the cardiac anatomy and associated malformations in clinical research.

**Objective:**

To determine the diagnostic value of peripheral mucin domain protein-3 (Tim-3), N-terminal pro-brain natriuretic peptide (NT proBNP), sestrin2 testing in patients with the left-to-right shunt congenital heart disease and heart failure.

**Methods:**

Fifty-two neonates with with left to right shunt congenital heart disease and 30 healthy neonates were enrolled. Blood samples were collected within 24 h of admission from newborns for determining the content of TiM-3, NT proBNP, and Sestrin2. Analyzing the ROC curve provided insight into the diagnostic accuracy. Both a Spearman’s rank correlation test and a logistic regression analysis were carried out.

**Results:**

TiM-3, NT proBNP, and Sestrin2 levels in peripheral blood were statistically different in the three groups (*P* < 0.05). There were significant differences in LVEF and LVFS among the three groups (*P* < 0.05). When used to diagnose heart failure in conjunction with left-to-right shunt congenital heart disease, TiM-3, NT proBNP, and Sestrin2 exhibited sensitivity of 58.3, 58.3, and 83.3%, respectively, and specificity of 85.0, 72.5, and 70.0%. ROC curve analysis showed that the AUCs of Tim-3, NT proBNP, and sestrin2 in predicting the outcome of left-to-right shunted congenital heart disease combined with heart failure were 0.744 (95% CI, 0.580 to 0.908), 0.608 (95% CI, 0.359 to 0.857), respectively 0.744 (95% CI 0.592 to 0.896).

**Conclusion:**

Tim-3, NT proBNP, and sestrin2 can accurately differentiate heart failure from non-combined heart failure from left-to-right shunt congenital heart disease.

## Introduction

Congenital heart disease is very common among birth defects and a leading cause of infant mortality or disability. Children with congenital heart disease are prone to serious complications such as heart failure and pulmonary infections. Shunt left-to-right congenital heart disease is distinguished by the presence of abnormal channels between the left and right chambers of the heart, resulting in a blood shunt from left-to-right. If not treated correctly and promptly, this type of congenital heart disease will eventually develop a “right-to-left” shunt, which is characterized by increased blood volume in the pulmonary circulation and decreased blood volume in the body circulation, and patients with this disorder are prone to recurrent respiratory tract infections [1]. Repeated respiratory infections can worsen pulmonary hypertension and impair cardiac function. Treatment options must be tailored to the individual condition. Patients with severe defects require immediate surgical treatment. The search for a noninvasive and accurate diagnostic method that correlates with cardiac anatomy and associated malformations is neededin clinical left-to-right shunt type congenital heart disease [2].

Mucin domain protein-3 (Tim-3), a Th1(T helper 1) lymphocyte marker molecule that regulates T lymphocyte subset function through corresponding ligands, is involved in the disease progression of autoimmune diseases and closely related to the development of congenital heart disease [3]. N-terminal pro-brain natriuretic peptide (NT proBNP) has a good diagnostic value for cardiac dysfunction. The diagnosis of heart failure in children is mainly based on clinical symptoms and signs [4]. Studies on the diagnostic value of NT proBNP for heart failure have mainly focused on adults, with fewer studies on heart failure in congenital heart disease [5]. Changes in BNP (B-type natriuretic peptide) levels before and after pediatric cardiac surgery are closely related to cardiac function. The detection of plasma NT proBNP levels in the perioperative period may serve as a basis for evaluating the condition in children. BNP is cleared mainly by binding to BNP clearance receptors. Human BNP is synthesized as a 134 amino acid precursor protein, which is cleaved into active BNP and inactive NT proBNP by enzymes produced upon secretion from cardiomyocytes. NT proBNP has been more widely used than BNP in clinics because of its long half-life and good performance at particularly ruling out heart failure in breathless patients. Sestrin2 is a stress-induced antioxidant protein that counteracts oxidative stress, inhibits ischemia-reperfusion injury, and regulates and controls thrombosis [6]. Sestrin2 is involved in the pathogenesis of acute ischemic stroke, and the cardiac remodeling process [7], but whether sestrin2 affects the development and progression of heart disease is unknown [8]. We assessed the diagnostic value of peripheral Tim-3, NT proBNP, and sestrin2 assays in left-to-right shunt congenital heart disease combined with heart failure, aiming to determine the presence or absence of heart failure in babies with left to right shunt.

## Materials and methods

### General information

All methods were carried out in accordance with relevant guidelines and regulations or Declaration of Helsinki. Fifty-two newborns with left-to-right shunt congenital heart disease admitted to our hospital from January 2020 to July 2022 were selected and divided into combined heart failure group with all left to right shunt (*n* = 16) and non-combined heart failure group (*n* = 36). The group with heart failure with all left to right shunt: 8 males and 8 females, age (4.34 ± 0.12) days; In the group without left-to-right shunt, 19 were male and 17 were female, age (4.25 ± 0.12 days). Inclusion criteria: (1) All cases with heart failurewere diagnosed by echo examination; (2) The diagnosis of heart failure complies with the diagnostic criteria of the 5th edition of Practical Neonatology:left heart failure with varying degrees of dyspnea and pulmonary rales, right heart failure with jugular vein signs, hepatomegaly, edema and heart failure with gallop rhythm and murmurs in the valvular region.; (3) Informed consent of guardians of newborns in this study; (4) Comply with Diagnostic Standard of Left-to-Right Shunt Congenital Heart Disease, 5th edition of Practical Neonatology. Exclusion criteria: (1) Combination of other cardiovascular diseases than Congenital heart disease; (2) Abnormal function of liver and kidney; (3) accompanied by diseases of the central nervous system and malignant tumors. At the same time, 30 healthy newborns in our hospital were selected as the control group, including 15 males and 15 females, aged (4.33 ± 0.11 days). Inclusion criteria: (1) All newborns were excluded from cardiovascular diseases by echo examination; (2) Informed consent of guardians of newborns in this study; (3) Exclude abnormal functions of liver and kidney, diseases of the central nervous system, and malignant tumors. There was no statistical difference in the general data of each group (*P* > 0.05). This study was approved by the Hospital Ethics Committee. Standard for normal neonates: ① Normal breathing: after birth, cry several times, and then start pulmonary respiration. In the first 2 weeks of life (when quiet), the breathing rate is 40–50 times per minute; ② Normal pulse: 120 ~ 140 times per minute at rest; ③ Normal temperature: the normal temperature of the newborn is 36 ~ 37.5 °C; ④ Normal weight: the weight of normal newborn is 3000-4000 g; ⑤ Normal stool: the baby’s stool is dark green, thick and tasteless, and is called meconium after 1–2 days of birth. After feeding, stool gradually turns yellow or golden (breast feeding) or light yellow (milk feeding). The baseline information was listed in Table 1.

**Table 1:** The baseline information

Group	*n*	Days	male/female
Control	30	4.33 ± 0.11	15/15
all left to right shunt with HF	16	4.34 ± 0.12	8/8
all left to right shunt without HF	36	4.25 ± 0.12	19/17
*P*		> 0.05	> 0.05

### Method

The control group received biochemical blood samples for th*e* routine newborn examination within 24 hours after being admitted to the same room of mother and infant to detect the levels of TiM-3, NT proBNP, and Sestrin 2.

TiM-3 detection method: TiM-3-PE 20 μl was added to the test tube after blood collection and anticoagulation respectively. Erythrocyte lysate was add after incubation at room temperature for 30 min. After continue incubation for 10 min, the superarant was collected through centrifugation. After washing with 200 μl PBS, the cells were resuspended cells and subjected to flow cytometer analysis (AAA-999, Beijing Zhonghua University Technology Co., Ltd.). The reagent is Tim3 recombinant antibody purchased from Aikangde Biomedical Technology (Suzhou) Co., Ltd. In these QC, both intra group and inter group CVs were < 10%. Data processing and quality control are completed by the clinical laboratory of our hospital.

Nt proBNP detection method: use the American VITROS that can be used for clinical diagnosis in our hospital® 5600 automatic biochemical immune analyzer and its supporting reagents. After instrument debugging and calibration, use the matched quality control to conduct quality control measurement and conduct sample testing after passing. Take 4 mL of whole blood, centrifugate and directly measure it with serum. All operations strictly follow the operating specifications of the instrument..

Sestrin2 detection method: after the blood is resting, take the supernatant and place it in the C1650R-230 V micro-high-speed refrigeration centrifuge provided by Leipter Scientific Instruments (Beijing) Co., Ltd. at 4 °C, 3000 r/min, 15 min centrifugal, 10 cm centrifugal radius. The supernatant was storedin the Freezerat − 80 °C. The serum Sestrin 2 level was detected by Sestrin-2 ELISA Kit (*Vitros*® Johnson & Johnson, *USA)*). The instrument is Gemini XPS full-wavelength enzymatic label provided by Mercury Molecular Instruments (Shanghai) Co., Ltd.. In these QCs, their intra- and inter-assay CVs were < 10–15%. Data processing and QC were done by our hospital clinical lab.

To determine left ventricular ejection fraction (LEVF), DC-28 color Doppler ultrasonic system. With the probe set to 3.5 ~ 5.0MHZ (Sheng Shida Medical Equipment Co., Ltd) was used to detect left ventricular ejection fraction (LEVF), left ventricular short axis shortening rate (LVFS), A peak E flow rate ratio (E/A).

### Statistical methods

SPSS22.0 statistical software was used to analyze and process the data. Data are presented as mean ± standard errors. Normal distribution of the data was demonstrated by the Kolmogorov–Smirnov test. Metrology data are expressed as mean standard deviation, the t-test is used for comparison betweentwo groups, and one-way ANOVA is used for comparison between three groups. Counting data used χ^2^ inspection; Pearson correlation test was used between dependent and independent variables. The sensitivity and specificity of TiM-3, NT-proBNP, and Sestrin2 detection in peripheral blood for left-to-right shunt congenital heart disease with heart failure was analyzed by ROC curve, with *P* < 0.05 as the significant difference.

## Results

### Comparison of Tim-3, NT proBNP, and sestrin2 levels in peripheral blood among the three groups

Both HF groups had higher levels of all 3 biomarkers compared to healthy newborns. HF + left to right shunt group was higher than HF alone group (*P* < 0.001), Table 2. The levels of Tim-3, NT proBNP, and sestrin2 in the peripheral blood of the three groups were statistically different (*P* < 0.05), and the levels of the above indicators in the combined HF group were higher than those in the non-combined and control groups, as shown in Table 2.

**Table 2:** Comparison of Tim-3, NT proBNP, and sestrin2 levels in peripheral blood among the three groups

Group	Case	TiM-3(%)	NT-proBNP(pg/ml)	Sestrin2(ng/mL)
left to right shunt with HF	16	20.06 ± 5.24	1236.1 ± 500.22	20.22 ± 5.18
left to right shunt without HF	36	16.22 ± 5.07	826.34 ± 200.27	15.09 ± 5.03
control group	30	5.06 ± 1.03	326.34 ± 100.17	7.06 ± 2.22
F-value		108.863	55.443	76.842
*P*-value		0.000	0.000	0.000

### Comparison of echocardiographic indexes among the three groups

There were significant differences in LVEF and LVFS among the three groups (*P* < 0.05), and the levels of the above indicators in the combined HF group were lower than those in the non-combined HF and control groups, while there was no statistical difference in E / A comparison among the three groups (*P* > 0.05), see Table 3.

**Table 3:** Comparison of echocardiographic indexes among the three groups

Group	Case	LVEF(%)	LVFS(%)	E/A
Combined HF group	16	56.96 ± 5.12	28.23 ± 5.09	1.59 ± 0.24
Uncombined HeartGroup	36	66.34 ± 6.13	33.66 ± 6.07	1.58 ± 0.27
Control group	30	69.27 ± 5.07	38.27 ± 5.04	1.60 ± 0.17
F-value		47.262	29.842	0.044
*P*-value		0.000	0.000	0.957

### Analysis of the correlation between Tim-3, NT proBNP, and sestrin2 levels in peripheral blood and the occurrence of heart failure in the child

The levels of Tim-3, NT proBNP, and sestrin2 in peripheral blood were all correlated with the occurrence of heart failure in the child (*P* < 0.05) and are shown in Table 4.

**Table 4:** Analysis of the correlation between Tim-3, NT proBNP, and sestrin2 levels in peripheral blood and the occurrence of heart failure in the child

Variable		Occurrence of heart failure in the child
TiM-3	R	0.382
	P	0.003
NT-proBNP	r	0.360
	P	0.004
Sestrin2	r	0.364
	P	0.004

### ROC analysis of peripheral Tim-3, NT proBNP, and sestrin2 in diagnosingthe combined occurrence of left-to-right shunt congenital heart disease with heart failure

**Fig. 1:**
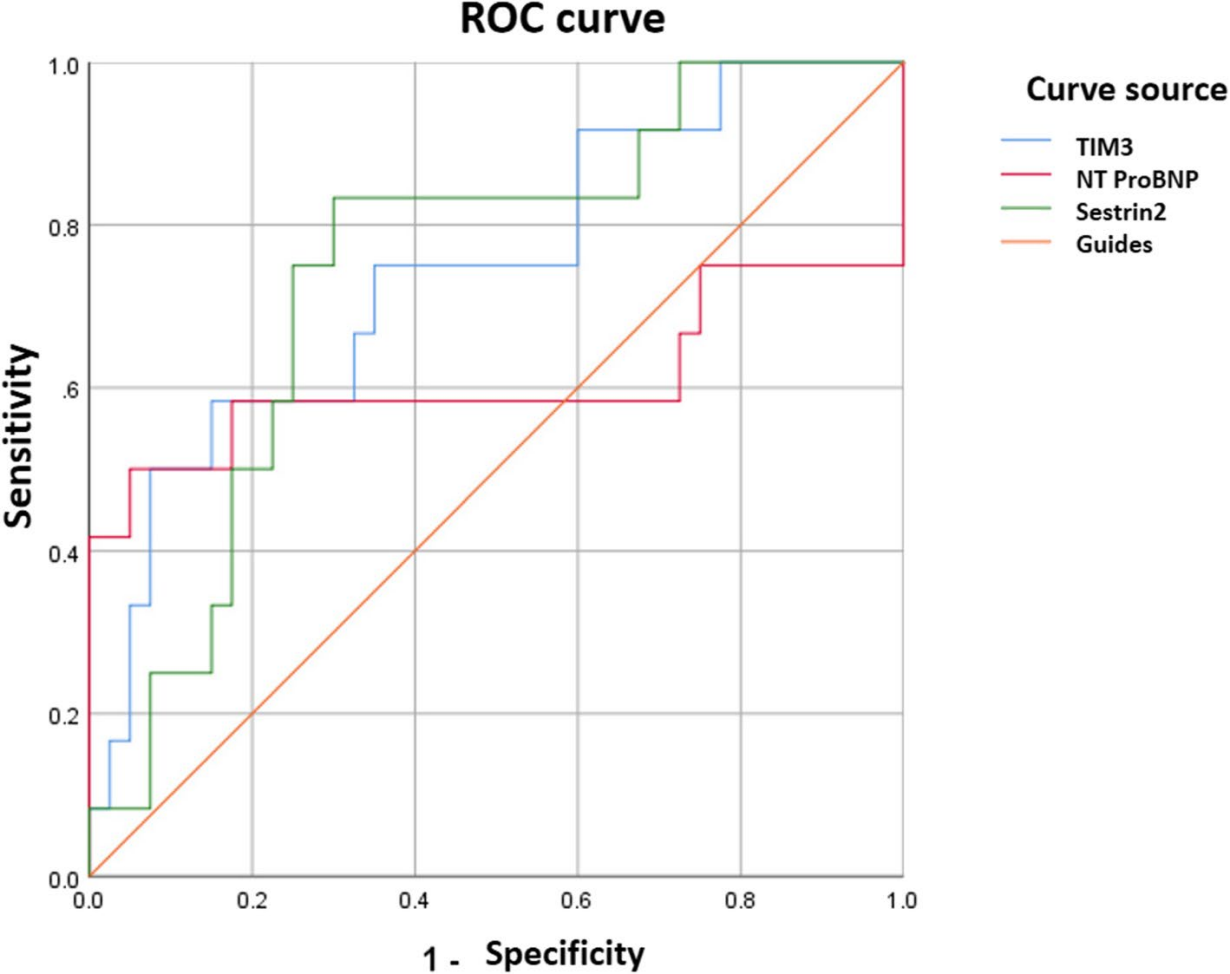
ROC curves of Tim-3, NT proBNP, sestrin2 in peripheral blood to diagnose the combinated heart failure with left-to-right shunt congenital heart disease

The sensitivity of Tim-3, NT proBNP, and sestrin2 in diagnosing the left-to-right shunt congenital heart disease with heart failure was 58.3, 58.3, 83.3%, respectively, and the specificity was 85.0, 72.5, 70.0%, respectively, shown in Table 5. Among them, Tim-3 and NT proBNP had a lower sensitivity compared with that of sestrin2. However, both them had higher specificity compared with that of sestrin2. Of note, the specificity of sestrin2 was of up to 70%. ROC curve analysis showed that the AUCs of Tim-3, NT proBNP, and sestrin2 in diagnosingthe left-to-right shunt congenital heart disease combined with heart failure were 0.744 (95% CI 0.580–0.908), 0.608 (95% CI 0.359–0.857), 0.744 (95% CI 0.592–0.896), respectively (Fig. 1).

**Table 5:** Effect of peripheral Tim-3, NT proBNP, sestrin2 in diagnosing the combinated heart failure with left-to-right shunt congenital heart disease with heart failure [% (n / N)]

Index	Cutoff	Sensitivity	Specificity	Accuracy	Positive predictive value	Sensitivity negative predictive value	Internal validation (95%CI)	AUC
**TIM-3(%)**	22.168	58.3	85.0	78.8	79.5	67.1	0.580–0.908	0.744
**NT-PROBNP(PG/ML)**	1110.178	58.3	72.5	69.2	68.2	63.5	0.359–0.857	0.608
**SESTRIN2(NG/ML)**	21.455	83.3	70.0	73.1	73.5	80.7	0.592–0.896	0.744

## Discussion

In general, congenital heart disease mainly refers to structural heart disease, among which left-to-right shunt type congenital heart disease is accounts up *to* 10%. Left-to-right shunt type congenital heart disease has special hemodynamic characteristics, which can cause congenital heart disease associated with pulmonary hypertension and can be complicated by pneumonia [9]. When pulmonary arterial pressure is elevated, it is more likely to be associated with pneumonia and pulmonary hypertension and pneumonia are high-risk factors for heart failure. The resulting vicious circle creates difficulties for the clinical diagnosis and treatment of left-to-right shunt type congenital heart disease [10]. Our data showed that plasma levels of Tim-3, NT proBNP, and sestrin2 are elevated in left to right shunt type congenital heart disease. Tim-3, NT proBNP, and sestrin2 have a ROC diagnostic value with an AUC value of 0.744, 0.608 and 0.744, respectively. And this is the first time, to our knowledge, that we present its diagnostic value of Tim-3, NT proBNP, and sestrin2 as an bio signature for predicting the combined heart failure of left-to-right shunt type congenital heart disease.

Tim-3, as an important cytokine secreted by Th1 cells, can bind to the ligand galectin-9 to regulate the body’s immune function. Which is an important negative feedback regulator of the immune response. Tim-3 is expressed on CD4 +, CD8 +, and other T cells, NK cells, and monocytes. The occurrence of congenital heart disease is closely related to autoimmune regulatory dysfunction, and Tim-3 plays an important role in this process [11]. Autoimmune development is encouraged by Tim-3 inhibition [12]. Heart failure’s underlying etiology or aggravation is increasingly understood to be autoimmune disease [13]. Yu and Haiwen [14] showed that the proportion of Tim-3 + CD4 + T cells was higher in heart failure patients than in healthy controls, and the proportion of Tim-3 + CD8 + T cells was also higher in heart failure patients than in healthy controls, indicating that the expression of T cell immunoglobulin and mucin domain-containing protein-3 (Tim-3) and its regulation on T cells were upregulated in chronic heart failure patients. However, whether it can be used as a diagnostic factor remained unknown. Our data showed that in newborns with left-to-right shunt congenital heart disease, particularly in those with concomitant heart failure, Tim-3 (peripheral) was much greater than in normal controls and increased. Tim3 in particular has high specificity (85.0%). In a word, Tim-3 might be a promising objective method for a rule out ofcombinedheart failure in left-to-right shunt congenital heart disease.

As biomarkers reflecting cardiac workload, ventricular remodeling, and cardiac function, BNP and NT proBNP a are released by elevated transmural pressure due to excessive pressure and volume expansion of cardiomyocytes [15]. BNP mainly exerts its effects by binding to natriuretic peptide receptors to form cyclic guanylate, including inhibiting the renin-angiotensin-aldosterone system, promoting renal sodium excretion diuresis; Suppression of ventricular remodeling; Dilated blood vessels; Inhibition of sympathetic activity and so on [16]. Ming Zhang et al. [17] found that preoperative plasma BNP was positively correlated with the occurrence of postoperative renal injury, hospitalization, and tracheal intubation time. These elevated levels of NT proBNP raise in response to heart damage. NT proBNP has high specificity (1110.178%). Cut-off value was 0.608 at 100 specificity. Therefore, NT proBNP will be better at ruling out HF.

Sestrin2 is a highly conserved stress-inducible protein molecule that inhibits the mammalian target of rapamycin (mTOR). However, whether they have diagnostic value remained unknown in combined heart failure with left-to-right shunt congenital heart disease. Our data showed that that It has been shown that sestrin2 exerts antioxidant effects through the hestrin2 / kelh like ECH associated protein 1 / nuclear erythroid related factor 2 pathway and thereby play an important role during the pathogenesis of heart disease [18]. Yingcun Liu et al. [19] showed that sestrin2 protein has a protective effect on hypoxia-reoxygenation-induced oxidative damage in cardiomyocytes, and the mechanism may be related to the activation of nuclear factor E2 related factor 2 / heme oxygenase-1 (HO-1) - related signaling pathway. Our data showed that Sestrin2 levels are markedly elevated in children with left-to-right shunt congenital heart disease compared to healthy controls, particularly in those with combined heart failure, indicating that high levels of sestrin2 may have an impact on how the condition progresses. The AUC value of diagnostic failure in left-to-right shunt congenital heart disease combined with heart failure outcome was 0.744, suggesting it has a better diagnostic value for the occurrence of left-to-right shunt congenital heart disease combined with heart failure.

In summary, the levels of Tim-3, NT proBNP, and sestrin2 in peripheral blood would be increased and LVEF and LVFS would be decreased in children with the left-to-right shunt congenital heart disease and heart failure, and the levels of Tim-3, NT proBNP and sestrin2 would have a better diagnostic value of left-to-right shunt congenital heart disease. The prospective, multi-centered, large-scale studies are warranted. Additionally, other clinical parameters in diagnostics of heart failure from non-combined heart failure from left-to-right shunt congenital heart disease should be considered.

## Data Availability

Due to concealment involving participants, privately anonymous datasets will be sent to by reasonable request corresponding author. If anyone wishes to obtain data from this study, they should contact Yan Teng.

## Abbreviations

Tim-3: Peripheral mucin domain protein-3
NT proBNP: N-terminal pro-brain natriuretic peptide
